# A Prospective Multicenter Study of the Chinese Scoring System for Hepatitis B Liver Failure

**DOI:** 10.3389/fmed.2021.751807

**Published:** 2021-11-02

**Authors:** Wu Zhe-bin, Lin Bing-liang, Peng Liang, Chen Zhi, Zhang Xin-xin, Tan De-ming, Ren Wan-hua, Wang Kai, Yan Xue-bin, Ke Wei-min, Zheng Yu-bao, Gao Zhi-liang

**Affiliations:** ^1^Department of Infectious Diseases, The Third Affiliated Hospital of Sun Yat-sen University, Guangzhou, China; ^2^The First Affiliated Hospital of Zhejiang University, Hangzhou, China; ^3^Shanghai Jiao Tong University Medical School Affiliated Ruijin Hospital, Shanghai, China; ^4^Xiangya Hospital, Central South University, Changsha, China; ^5^Department of Infectious Diseases, Shandong Provincial Hospital, Jinan, China; ^6^Department of Infectious Diseases, Shandong University Qilu Hospital, Jinan, China; ^7^The Affiliated Hospital of Xuzhou Medical University, Xuzhou, China

**Keywords:** hepatitis B, liver failure, scoring system, end-stage liver disease model, assessing

## Abstract

**Objective:** To evaluate the clinical utility of a Chinese scoring system for hepatitis B liver failure in a prospective and multicenter study.

**Methods:** Clinical data for 1,143 patients with hepatitis B liver failure who had been followed up for a minimum of 6 months were collected from seven liver disease centers across China. The disease severity and prognosis for the patients were predicted using the Chinese scoring system and compared to those predicted with the model for end-stage liver disease (MELD) score, MELD-Na score, and Child-Turcotte-Pugh (CTP) score.

**Results:** The Chinese scoring system was more effective at predicting the outcomes of survival and mortality than the MELD score. In the peak disease stage, the area under the receiver operating characteristic curve for the Chinese scoring system was 0.954, significantly higher than that (0.896) for the MELD scoring system (*P* < 0.001). The positive prediction at 30, 90, and 180 days with the Chinese scoring system was 0.764 (95% CI: 0.714–0.808), 0.731 (95% CI: 0.694–0.769), and 0.724 (95% CI: 0.679–0.765), also significantly higher than that with the MELD, MELD-Na, and CTP scores (*P* < 0.001). In addition, the Chinese scoring system was superior to the MELD, MELD-Na, and CTP scores (*P* < 0.001) at predicting the prognosis of patients with hepatitis B liver failure at both 30 and 180 days.

**Conclusion:** The Chinese scoring system demonstrated superior performance to the three established scoring systems in assessing the severity and outcomes of hepatitis B liver failure in this cohort.

## Introduction

Liver failure is a severe complication in which the liver fails to perform basic functions due to severe or massive liver injury, which also disrupts the vital functions of other organs and systems. It typically manifests as jaundice, hepatic encephalopathy, and coagulopathy (1, 2). The mortality rate of liver failure may be as high as 70% (3). Hepatologists must be able to assess and predict the outcomes of liver failure accurately and early in patients, especially to determine if and when liver transplantation is required. Dynamic changes in the conditions of a patient should also be closely monitored and followed, as timely prevention and treatment of complications significantly impact the clinical outcomes. The models for end-stage liver disease (MELD), MELD-Na, and Child-Turcotte-Pugh (CTP) scores are three established systems used to numerally grade the severity of liver failure in patients, which can reveal the probability for favorable or non-favorable outcomes (4, 5). All three model systems were established by assessing patients with liver failure mainly caused by hepatitis C virus (HCV) infection and alcoholic liver disease. However, liver failure caused by hepatitis B virus (HBV) infection is characterized by acute onset, severe liver injury, and high mortality, with distinct pathogenesis and clinical characteristics from those caused by HCV and alcohol. The three model systems can be expanded or modified to better grade HBV liver failure. We previously established a new scoring system known as the Chinese scoring system and its performance and utility were evaluated for patients with hepatitis B liver failure in a retrospective study (6–9). To further investigate the utility of this new scoring system in grading the severity of hepatitis B liver failure and predicting outcomes, we conducted this prospective study at seven liver disease centers across China.

## Materials and Methods

### Patients

#### Enrollment

We selected seven liver disease centers across China (infection department of the Third Affiliated Hospital of Sun Yat-sen University, infection department of the First Affiliated Hospital of Zhejiang University, Shanghai Ruijin Hospital, Xiangya Hospital of Central South University, Qilu Hospital of Shandong University, Shandong Provincial Hospital, and Xuzhou Medical College) with a high reputation in the country, high diagnosis and treatment capabilities in the field of liver disease, and more than 40 beds as the enrollment unit. The sample size was determined using a single population proportion formula by considering a 5% margin of error, 95% confidence level, 50% case fatality rate of liver failure in previous studies, 5% non-response rate, and 1.5 design effect. The resulting total sample size was 1,078. To account for potential dropouts, the total sample size was fixed to 1,200 and proportioned to the selected participating hospitals based on their clients' size: infection department of the Third Affiliated Hospital of Sun Yat-sen University (*n* = 400), infection department of the First Affiliated Hospital of Zhejiang University (*n* = 200), Shanghai Ruijin Hospital (*n* = 200), Xiangya Hospital of Central South University (*n* = 100), Qilu Hospital of Shandong University (*n* = 100), Shandong Provincial Hospital (*n* = 100), and Xuzhou Medical College (*n* = 100).

Between October 2013 and June 2016, a total of 1,143 chronic hepatitis B inpatients with liver failure (acute-on-chronic liver failure, ACLF) were enrolled from seven liver disease centers. Among them, 936 were men, 207 were women, and the average age was 43.4 ± 12.6 (ranged from 18 to 65). The patients were followed up for >6 months, during which 464 died and 680 survived. This study was registered in the Clinical Trials Registry (registration number: NCT01961440, https://clinicaltrials.gov/).

HBV-related ACLF diagnosis for all patients followed the criteria established by the 18th Asia-Pacific Association of Liver Research consensus on chronic-acute liver failure (10). The criteria include a history of chronic hepatitis B and an acute flare-up of liver injury with clinical manifestations of jaundice (total bilirubin ≥ 85 μmol/l) and coagulation disorder (prothrombin time international standardized ratio ≥ 1.5), ascites, and/or hepatic encephalopathy within 4 weeks.

#### Inclusion Criteria

Male or female patients aged 18–60; HBsAg-positive history > 6 months, HBV DNA-positive (≥20 IU/ml); HBeAg-positive or negative; persistent hepatitis symptoms of fatigue, anorexia, abdominal distention, or yellow urine; gradual aggravation of jaundice over a short period; total serum bilirubin ≥85 μmol/l or daily elevation ≥17.1 μmol/l; abnormal coagulation function; and the international standardized ratio of prothrombin time ≥1.5 were included.

#### Exclusion Criteria

Patients were excluded if they: (1) had other hepatitis virus infection; (2) had HIV infection, biliary, alcoholic liver, or autoimmune liver diseases; drug poisoning, liver tumors, or were undergoing liver transplantation, renal insufficiency, or long-term anticoagulant therapy related to renal diseases.

### Observations and Follow-Up Endpoints

Clinical information and test findings for all patients at and after admission were collected weekly. The clinical information mainly consisted of the stages of hepatic encephalopathy. Laboratory findings included serum total bilirubin, albumin, creatinine, prothrombin time, prothrombin time international normalized ratio (INR), serum sodium ion concentration, liver size (B-mode ultrasound measurement), ascites, and pleural effusion (B-ultrasound measurement), and also infection (peripheral white blood cell count, neutrophil ratio, and chest inflammation images). The endpoint of 180 days of follow-up was used to determine the survival rate. The death count included patients that rejected rescue treatment and were discharged from the hospital and patients that died during the hospital stay or within 180 days of follow-up.

### Score Calculation

#### MELD Score

Since all subjects had hepatitis B-related liver failure, the MELD score was calculated as 3.8 × loge (serum bilirubin μmol/l × 0.058) + 1.2 × loge (prothrombin time INR) + 9.6 × loge (serum creatinine μmol/l × 0.011) + 6.4 (6).

#### MELD-Na

This score was calculated as MELD + 1.59 × (135-Na^+^), wherein serum Na^+^ levels ≥ 135 mmol/l were treated as 135, ≤ 120 as 120 mmol/l, and between 120 and 135 mmol/l as the specific value (7).

#### CTP Score

This score was calculated using the scoring standards for five indexes, i.e., grade of hepatic encephalopathy, ascites, total bilirubin, albumin, and prolonged prothrombin time. A score of 1, 2, or 3 was assigned to each index to reflect the severity of each condition, and the CTP score was the sum of the five indexes (8).

#### Chinese Scoring System

This system consists of seven clinical indicators: prothrombin activity (PTA), serum creatinine, hepatic encephalopathy, serum total bilirubin, and liver size (B-ultrasonic measurement: oblique diameter of the right lobe of the liver: The standard measurement section is the oblique section of the right subcostal liver where the right hepatic vein and the middle hepatic vein merge into the inferior vena cava. The measurement points are placed at the liver envelope on the anterior and posterior edges of the right lobe to measure the maximum vertical distance; thickness of the right liver lobe: The largest section of the right lobe of the liver in the fifth or sixth intercostal space is the standard measurement section. The measurement points are placed at the liver envelope on the anterior and posterior edges of the right lobe to measure the maximum vertical distance), ascites/pleural fluid (B-ultrasonic measurement: When lying supine, measure the depth of the anterior liver, liver and kidney recesses, splenic fossa, right abdomen, and pelvic ascites, and take the largest value for score), volume, and infection (peripheral blood leukocyte count, neutrophil ratio, and chest inflammation image). A score of 1, 2, 3, or 4 was assigned to each indicator to reflect the severity, and the sum of the seven indicator scores was used to assess the severity of the overall condition (see Table 1 for details). Unified operation method of ultrasound.

**Table 1:** Assigned scores of the Chinese scoring system.

**Scoring** **(score)**	**Hepatic** **encephalopathy** **(Stage)➄**	**Total bilirubin** **(μmol/L)**	**The maximum** **depth of** **ascites (mm)➁**	**Activity of** **PT (%)**	**Right hepatic** **oblique diameter/** **thickness (mm)➂**	**Creatinine** **(μmol/L)**	**Infection** **(depend on WBC 10^**9**^/L)➃**
1	I	≥10~20ULN➀	>0~40	30~ <40	Oblique diameter ≥ 120 or Thickness ≥ 110	>1.0~1.1ULN	WBC>10~15 Or N>70%~80%
2	II	>20~30ULN	>40~80	20~ <30	Oblique diameter 110 ~ <120 or Thickness 100~ <110	>1.1~1.2ULN	WBC>15~20 Or N>80%~90%
3	III	>30~40ULN	>80	10~ <20	Oblique diameter100~ <110 or Thickness 90~ <100	>1.2~1.3ULN	WBC>20 Or N>90%
4	IV	>40ULN	>80 + one or both side pleural fluid	<10	Oblique diameter <100 or Thickness <90	>1.3ULN	Inflammation manifestation of lung

注: ➀ *ULN: Upper limit of normal.*

➁ *Score of ascites: Regardless of the amount of ascites, the presence of unilateral or bilateral pleural fluid is counted as 4 points.*

➂ *Liver size scoring: If the right liver oblique diameter and thickness are measured at the same time on the ultrasound image, the high-scoring scoring results are taken.*

➃ *Infection score: The standard of 4 points is the imaging changes of pulmonary inflammation, regardless of the white blood cells and neutrophil counted as 4 points.*

➄ *HE grade 1: Trivial and mild clinical signs, such as mild cognitive impairment, decreased attention, sleep disorders (insomnia and sleep inversion), euphoria, or depression; Grade 2: Marked personality or behavioral changes, lethargy or apathy, slight orientation abnormality (time and orientation), decreased mathematical ability, dyskinesia, or unclear speech; Grade 3: Marked dysfunction (time and spatial orientation), abnormal behavior, semi-coma to coma, but responsive; Grade 4: Coma (no response to speech and external stimuli)*.

### Statistical Analysis

(1) All numeral data were expressed as mean ± SD (±s) and the difference was computed using the *f*-test. (2) The count data were expressed as a percentage (%), and the difference was assessed with the χ^2^ test. (3) Assessments of the short-term and long-term prognoses for patients with hepatitis B liver failure using the Chinese scoring system, MELD score, MELD-Na score, and CTP score were compared using receiver operating characteristic (ROC) curves and the ROC area under the curve (AUC). AUC > 0.7 was deemed to be of clinical utility and >0.8 to be of good prediction accuracy. The AUC values were compared using normal *Z*-tests and the ROC curve sensitivity and specificity were used to determine the best cut-off values for the score and the Youden's index. SPSS l8.0 statistical software (IBM SPSS Inc., Chicago, USA) was used for all analyses. *P* <0.05 was considered statistically significant.

## Results

### Demographic Features and Groups

A total of 1,143 patients with hepatitis B liver failure were enrolled in this study. Among them, 936 were men and 207 were women, and the mean age was 43.4 ± 12.6 years old (ranged between 18 and 65). A total of 463 patients died while the remaining 680 survived (Table 2).

**Table 2:** Baseline characteristics of included patients at admission.

**Parameters**	**Death group** **(n = 463)**	**Survival group** **(n = 680)**	**t/x2 value**	***P***
Age (year)	45.38 ± 11.73	40.06 ± 10.47	1.84	0.12
Males (%)	364/99	572/108	1.99	0.06
WBC (×109/L)	7.82 ± 3.54	6.98 ± 3.72	1.25	0.38
ALT (U/L)	568.47 ± 376.94	629.76 ± 504.57	1.56	0.28
Albumin (g/L)	31.64 ± 3.56	32.85 ± 4.02	0.98	0.47
TB (lmol/L)	428.75 ± 284.63	353.96 ± 249.66	3.45	<0.001
PTA (%)	29.86 ± 11.58	34.38 ± 10.85	4.78	<0.001
INR	3.41 ± 0.68	2.83 ± 0.62	4.27	<0.001
BUN/(mmol/L)	5.84 ± 3.68	5.22 ± 3.76	1.96	0.08
CR/(μmol/L)	82.37 ± 54.16	67.47 ± 44.85	1.92	0.09
HBV DNA, median (range), Log10 IU/ml	6.89 (4.30-8.86)	6.62(5.02-8.64)	1.89	0.16
Without cirrhosis, *n*(%)	296(63.93)	258(37.94)	8.94	<0.001
Encephalopathy (%)	16.58	9.73	10.76	<0.001
HRS (%)	8.23	5.13	2.95	<0.001
SBP (%)	54.32	31.06	6.83	<0.001
Ascites (%)	67.43	48.14	3.12	<0.001
MELD	40.52 ± 6.83	26.84 ± 6.18	16.83	<0.001
MELD-Na	44.76 ± 7.83	28.62 ± 5.93	19.05	<0.001
CTP	11.87 ± 1.68	10.56 ± 1.47	2.01	0.04
Chinese scoring system	17.53 ± 3.92	8.46 ± 3.28	22.18	<0.001

### The Cutoff Value and Distribution Range for the Chinese Scoring System and the MELD Score to Separate the Patients That Survived From Those That Did Not

**Figure 1:**
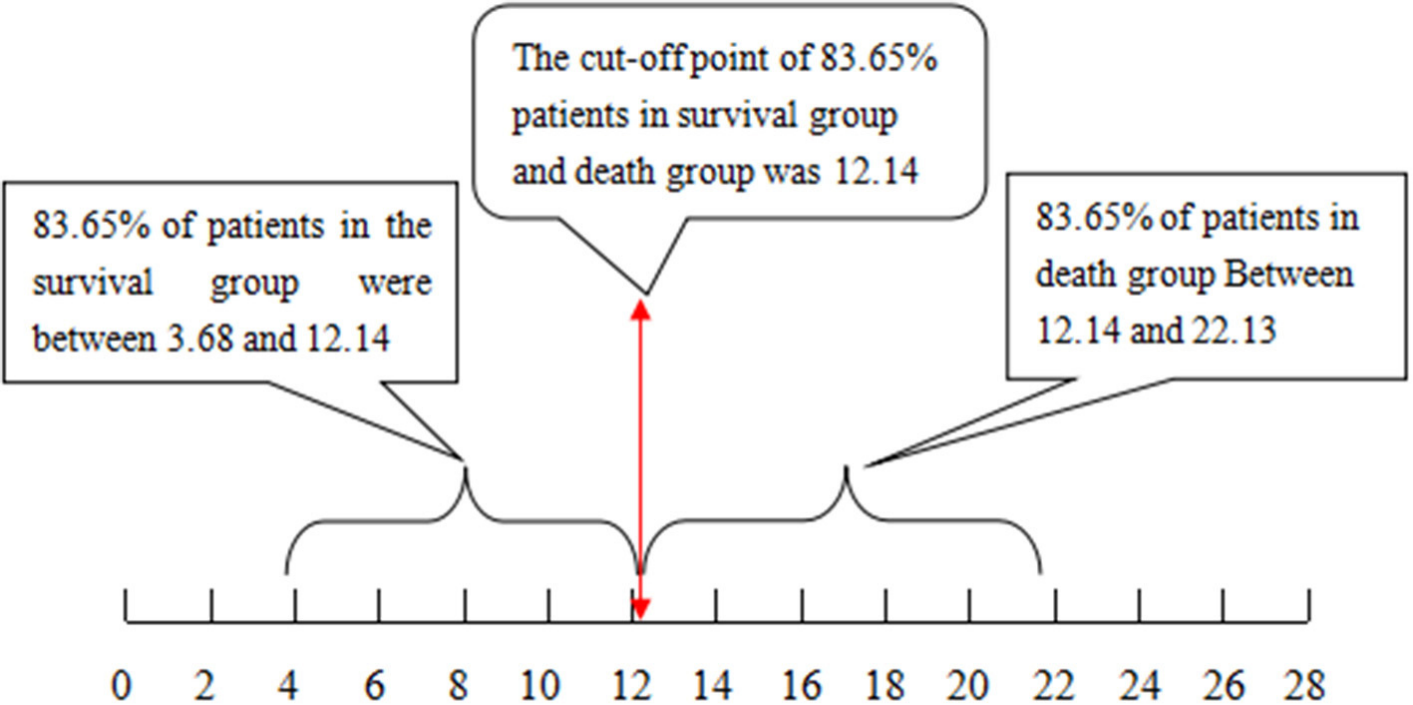
The cutoff value and distribution range for the Chinese scoring system to separate the patients that survived from those that did not.

**Figure 2:**
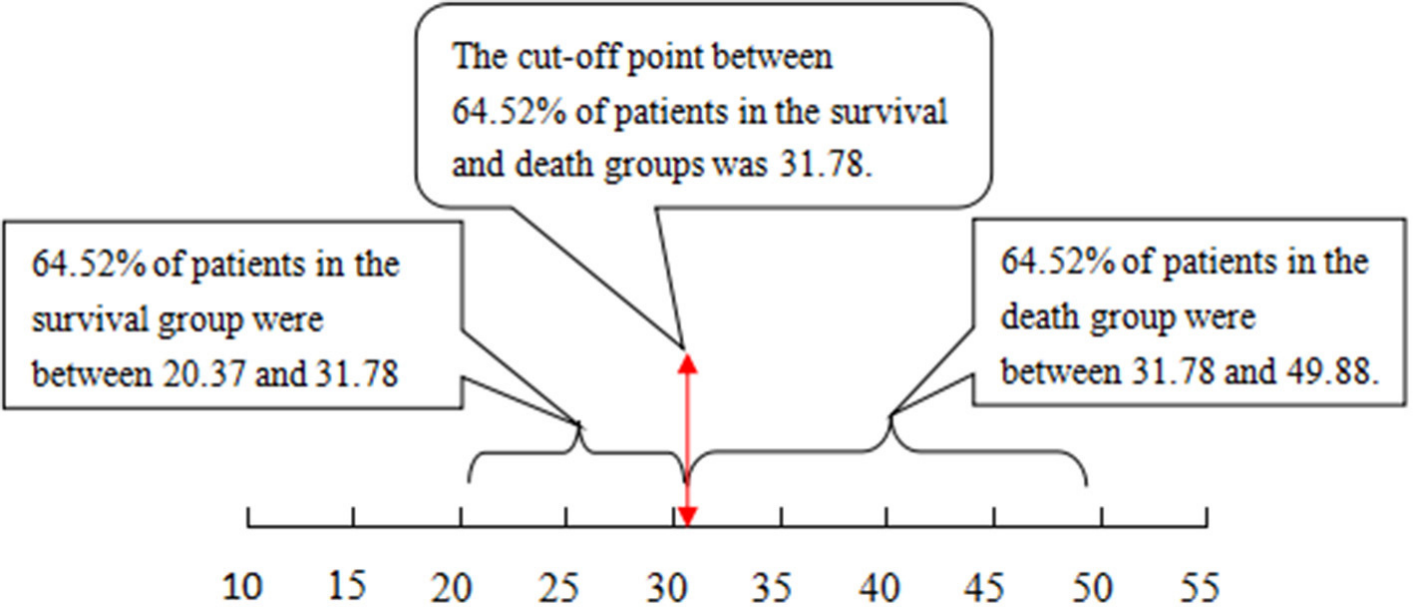
The cutoff value and distribution range for the MELD score to separate the patients that survived from those that did not. MELD, model for end-stage liver disease.

The cutoff value of 12.14 with the Chinese scoring system separated 83.65% of patients in the survival and death groups, i.e., 83.65% of patients in the survival group scored between 3.68 and 12.14 points, while 83.65% of patients in the death group scored between 12.14 and 22.13 points. With the MELD score, the cutoff value was 31.78; 64.52% of patients in the survival group scored between 20.37 and 31.78 points, and 64.52% of patients in the death group scored between 31.78 and 49.88 points (see Figures 1, 2, *P* < 0.01).

### Comparison of the Performance to Grade the Severity at Peak Disease With the Chinese Scoring System and MELD Scoring System

**Figure 3:**
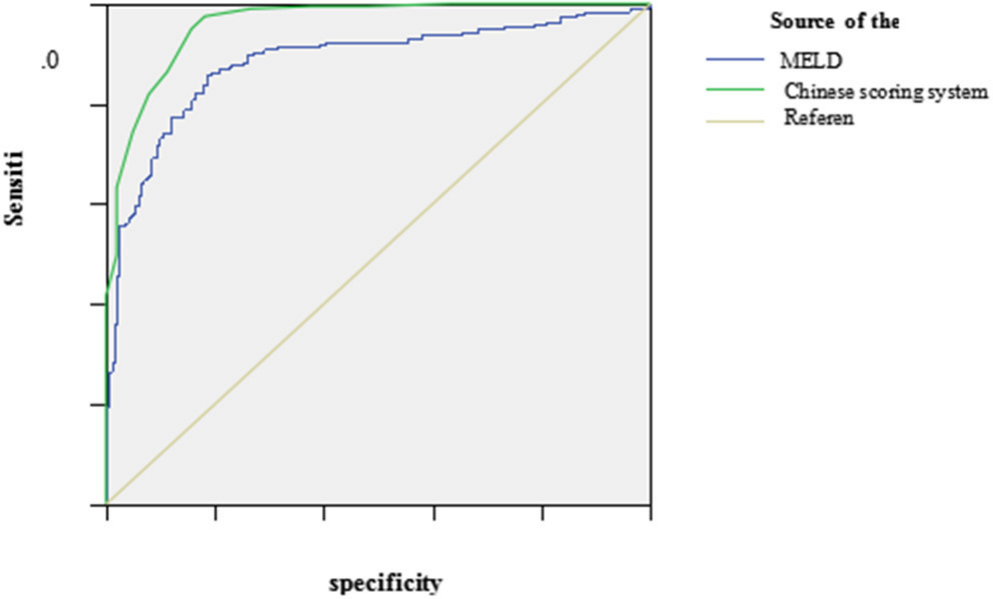
The ROC curve of the Chinese scoring system and MELD scoring system at the peak disease. MELD, model for end-stage liver disease; ROC, receiver operating characteristic.

The ROC curve was plotted using the total scores. The area under the ROC curve for the Chinese scoring system was 0.954 (95% CI: 0.943–0.971) and the standard error was 0.013, with *P* < 0.001, suggesting good prediction performance. The area under the ROC curve for the MELD score was 0.896 (95% CI: 0.881–0.924), indicating less effectiveness than the Chinese score system (see Figure 3 and Table 3).

**Table 3:** Comparison of the performance to grade the severity at the peak disease by Chinese scoring system and MELD scoring system.

	**AUROC (95% CI)**	***p*-value vs. CSS-LFB**
CSS-LFB	0.954 (0.943–0.971)	
MELDs	0.896 (0.881–0.924)	<0.001

### The Ability to Predict and Differentiate Disease Outcomes at Different Times With Different Scoring Systems

The predictive values for the outcomes on days 30, 90, and 180 after discharge using the Chinese scoring system were 0.764 (95% CI: 0.714–0.808), 0.731 (95% CI: 0.694–0.769), and 0.724 (95% CI: 0.679–0.765), respectively, which were significantly higher than those for the other three scoring systems (see Table 4, *P* < 0.001).

**Table 4:** The ability to predict and differentiate disease outcomes at different times with different scoring systems.

	**CSS-LFB** **C-index (95% CI)**	**MELD** **C-index (95% CI)**	**MELD-Na** **C-index (95% CI)**	**Child-Pugh** **C-index (95% CI)**
30-day mortality	0.764 (0.714–0.808)	0.684 (0.632–0.743)	0.685 (0.634–0.738)	0.664 (0.612–0.725)
*p*-value vs. CSS		<0.001	<0.001	<0.001
90-day mortality	0.731 (0.694–0.769)	0.657 (0.612–0.713)	0.662 (0.616–0.712)	0.656(0.607–0.704)
*p*-value vs. CSS		<0.001	<0.001	<0.001
180-day mortality	0.724 (0.679–0.765)	0.648 (0.604–0.698)	0.655 (0.607–0.699)	0.642 (0.593–0.691)
*p*-value vs. CSS		<0.001	<0.001	<0.001

### Comparison of the Performance Predicting Short-Term (30 Days) Prognosis for Patients With Hepatitis B Liver Failure Using the Chinese Scoring System, MELD Score, MELD-Na Score, and CTP Score

**Figure 4:**
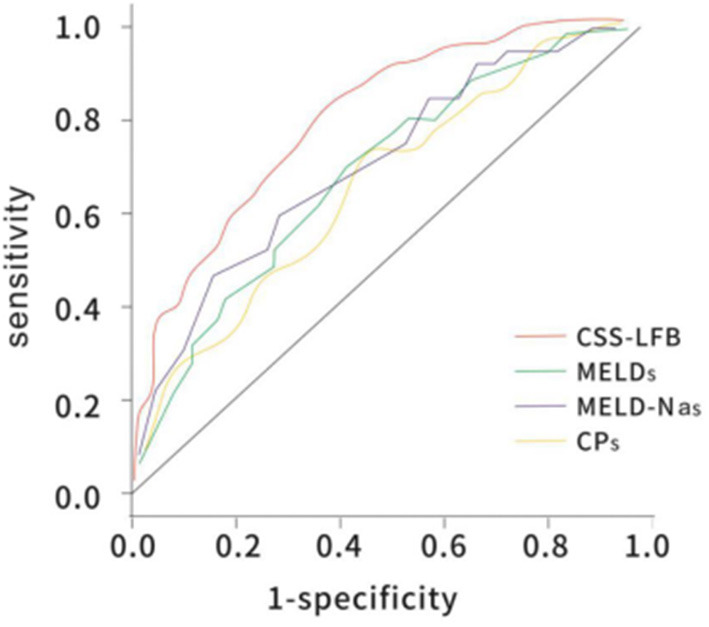
The ROC curve of predicting short-term (30 days) prognosis of patients with hepatitis B liver failure among the Chinese scoring system, MELD score, MELD-Na scoring, and CTP scoring. CTP, Child-Turcotte-Pugh; MELD, model for end-stage liver disease; ROC, receiver operating characteristic.

The area under the ROC curve with the total score from the Chinese scoring system was 0.783 (95% CI: 0.732–0.865) and the standard error was 0.026, with *P* < 0.001, suggesting good predictive performance. The areas under the ROC curves for the MELD score, MELD-Na score, and CTP score were 0.706 (95% CI: 0.623–0.778), 0.716 (95% CI: 0.644–0.779), and 0.686 (95% CI: 0.623–0.767), respectively, which were significantly lower than that for the Chinese scoring system (see Figure 4 and Table 5).

**Table 5:** Comparison of the area under the ROC curve of predicting short-term (30 days) prognosis of patients with hepatitis B liver failure among Chinese scoring system, MELD score, MELD-Na scoring, and CTP scoring.

	**AUROC (95% CI)**	***p*-value vs. CSS-LFB**
CSS-LFB	0.783 (0.732–0.865)	
MELDs	0.706 (0.623–0.778)	0.0089
MELD-Nas	0.716 (0.644–0.779)	0.0097
CPs	0.686 (0.623–0.767)	0.0075

### Comparison of the Performance Predicting Long-Term (180 Days) Prognosis for Patients With Hepatitis B Liver Failure Using the Chinese Scoring System, MELD Score, MELD-Na Score, and CTP Score

**Figure 5:**
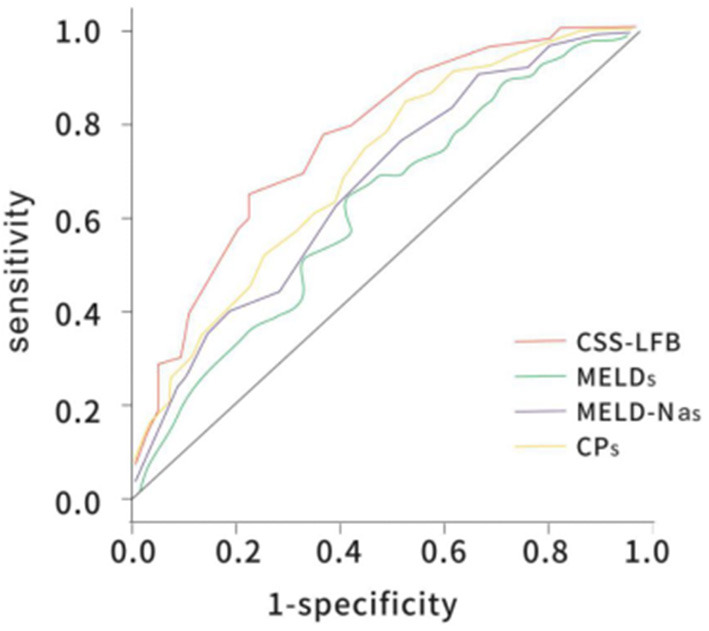
The ROC curve of predicting long-term (180 days) prognosis of patients with hepatitis B liver failure among the Chinese scoring system, MELD score, MELD-Na scoring, and CTP scoring. CTP, Child-Turcotte-Pugh; MELD, model for end-stage liver disease; ROC, receiver operating characteristic.

The area under the ROC curve with the total score from the Chinese scoring system was 0.748 (95% CI: 0.692–0.837) and the standard error was 0.022, with *P* < 0.001, suggesting good predictive performance. The areas under the ROC curves for the MELD score, MELD-Na score, and CTP score were 0.657 (95% CI: 0.588–0.725), 0.676 (95% CI: 0.605–0.743), and 0.682 (95% CI: 0.621–0.752), respectively, which were significantly lower than that for the Chinese scoring system (see Figure 5 and Table 6).

**Table 6:** Comparison of the area under the ROC curve of predicting long-term (180 days) prognosis of patients with hepatitis B liver failure among Chinese scoring system, MELD score, MELD-Na scoring, and CTP scoring.

	**AUROC (95% CI)**	***p*-value vs. CSS-LFB**
CSS-LFB	0.748 (0.692–0.837)	
MELDs	0.657 (0.588–0.725)	0.0014
MELD-Nas	0.676 (0.605–0.743)	0.0082
CPs	0.682 (0.621–0.752)	0.0012

## Discussion

MELD, MELD-Na, and CTP scores have been established to address the need to accurately identify patients with liver failure who require liver transplants. All three systems are widely accepted in the clinical setting. However, these models were constructed using clinical data largely from patients with liver failure in Europe and the United States where the major etiologies for liver failure are alcohol, drugs, hepatitis C, and cholestasis. In contrast, hepatitis B represents the dominant etiology for liver failure in China and is responsible for 85% of liver failure cases in the country (11). Therefore, these three models (12, 13) may not completely fit Chinese patients with liver failure. As noted by Lin Xianfeng et al. (14), the MELD score failed to accurately predict the prognosis of 327 Chinese patients with hepatitis B liver failure. One factor that may have affected this result is that patients with HBV liver failure do not respond well to comprehensive treatment and often need an artificial liver, liver transplant, or other treatment urgently. The other factor is that chronic HBV infection may exhaust the immunity of patients, leading to severe complications. A new system that considers these factors is required (15).

Child-Turcotte-Pugh (CTP) score has been widely employed in managing patients with liver diseases because of its simplicity and ease of calculation. However, it is often hindered by a lack of consistency as CTP is mainly based on albumin level and prothrombin time, both of which can be masked if exogenous albumin and plasma products are infused. In addition, hepatic encephalopathy and ascites measurement can be subjective and variable. CTP only consists of three grades with a narrow score range of 5–15, which limits the ability to differentiate. Patients with the same score may differ greatly in the severity of the liver disease.

Although the MELD and MELD-Na scoring systems are recognized as easy to use, reproducible, and accurate in clinical applications, they bear an inherent limitation because they only include bilirubin, coagulation function, renal function, and etiology, while excluding the complications of liver failure (infection, hemorrhage, hepatic encephalopathy, brain edema, or ascites). The Na concentration in the MELD-Na scoring system can be easily affected by insufficient intake, diuretics, or sodium pump failure (12, 16–20). A study conducted in China (21) showed that the AUC of MELD and MELD-Na scores for the 12-week outcomes of patients with hepatitis B-related liver failure was only 0.731, 0.735, and 0.773, respectively, and the sensitivity was below 0.7.

This prospective study conducted at seven large liver disease centers across China showed that the Chinese scoring system better distinguished patients that survived from those who did not, in comparison to MELD (*P* < 0.001). The area under the ROC curve for the Chinese scoring system was 0.954 in the peak disease stage, suggesting a stronger ability to grade the severity of the liver failure. The AUC for the MELD scoring system was 0.896, which was relatively high, but significantly lower than that for the new scoring system (*P* < 0.001). We also found that the efficiency in predicting outcomes on the 30th, 90th, and 180th days after discharge with the Chinese scoring system was significantly higher than that with MELD, MELD-Na, and CTP (*P* < 0.001).

Massive or submassive liver injury following a flare-up of HBV replication or HBV reactivation compromises synthetic, metabolic, and detoxification functions in the liver, leading to reduced albumin levels and ascites, hyperbilirubinemia, decreased PTA, increased international standard ratio of prothrombin (INR), and hepatic encephalopathy. In addition, a severely injured liver may also hurt Kupffer cell function and reduce complement levels that may limit the anti-infective capacity. The accumulation of toxins and a decrease in renal blood flow may facilitate hyperbilirubinemia, renal function insufficiency, or even renal failure. In theory, the severity of liver failure among different individuals can be indicated by biomarkers that reflect critical alterations in the pathophysiology of liver failure. Thus, we formulated this new scoring system to improve severity grading and outcome prediction. A total of seven clinical indicators of hepatitis B liver failure are included in the Chinese scoring system, consisting of not only objective indicators such as Cr, total bilirubin, PTA, and liver size but also the complications of hepatic encephalopathy, ascites with pleural effusion, and pulmonary infection, which reflect the pathophysiological changes resulting from hepatitis B liver failure. The score boundary point was confirmed using the interactive chi-squared test, guided by the principle that emphasizes simplicity and clarity. Each index is graded between 0 and 4 points, allowing a high degree of differentiation that increases prediction accuracy. This new scoring system utilizes common clinical indicators, which can be routinely ordered to allow easy and wide applications.

This new scoring system demonstrated superior performance in grading the severity and predicting the outcomes of HBV liver failure in this cohort compared to the three established scoring systems, representing progress in improving the management of HBV liver failure. However, our findings will need further verification in larger cohorts that include not only patients with HBV liver failure but also those with liver failure with other etiologies.

## Data Availability Statement

The raw data supporting the conclusions of this article will be made available by the authors, without undue reservation.

## Ethics Statement

The studies involving human participants were reviewed and approved by Clinical Trials registry (registration number: NCT01961440, https://clinicaltrials.gov/). The patients/participants provided their written informed consent to participate in this study.

## Author Contributions

WZ-b performed the case summary and statistical analysis and wrote the manuscript. LB-l and PL were responsible for statistical analysis. CZ, ZX-x, TD-m, RW-h, WK, and YX-b were responsible for case enrollment and data collection in other subcenters. KW-m designed this scoring system. ZY-b was responsible for the whole quality of the study. GZ-l was responsible for research design and the whole quality of the study. All authors contributed to the article and approved the submitted version.

## Funding

This work was supported by the National Science and Technology Major Project (2018ZX10302204-002) and the National Natural Science Foundation of China (81672701).

## Conflict of Interest

The authors declare that the research was conducted in the absence of any commercial or financial relationships that could be construed as a potential conflict of interest.

## Publisher's Note

All claims expressed in this article are solely those of the authors and do not necessarily represent those of their affiliated organizations, or those of the publisher, the editors and the reviewers. Any product that may be evaluated in this article, or claim that may be made by its manufacturer, is not guaranteed or endorsed by the publisher.
